# Phenolic Chemical Defense Contributes to Resistance against Multiple Soybean Cyst Nematode Populations in Wild Soybean

**DOI:** 10.64898/2026.09.12.751195

**Published:** 2026-09-17

**Authors:** Hengyou Zhang, Neha Mittal, Xu Li, Zenglu Li, Chunying Li, Han-Yi Chen, Eric Davis, Changbao Li, Qijian Song, Yong-qiang Charles An, Bao-Hua Song

## Abstract

Soybean cyst nematode ( *Heterodera glycines* , SCN) is one of the most damaging pathogens of soybean worldwide. Virulence diversity among SCN populations and reliance on a limited number of resistance sources present major challenges for durable SCN management. Here, we investigated a wild soybean ( *Glycine soja* ) genotype (WsR), that is resistant to two SCN populations, race 2 (HG type 1.2.5.7) and race 5 (HG type 2.5.7), and compared its transcriptional and metabolic responses with those of the susceptible genotype (WsS). WsR exhibited a substantially stronger defense-associated transcriptional response to both SCN populations, including preferential induction of genes associated with Ca²⁺/calmodulin and salicylic acid signaling. Integration of transcriptomic and time-resolved metabolomic analyses revealed convergence on phenolic metabolism, with enhanced accumulation of phenolic acids, flavonoids, and isoflavonoids in resistant WsR. Genes involved in phenolic biosynthesis and modification were also preferentially induced in WsR, linking transcriptional reprogramming with the observed metabolic response. Importantly, two resistance-associated phenolic compounds, 4-hydroxybenzaldehyde and 2,3-dihydroxybenzoic acid, directly increased mortality of SCN second-stage juveniles from both populations in a concentration-dependent manner. By connecting resistance-associated transcriptional responses and metabolic reprogramming with direct activity of specific phenolic compounds against SCN, our study provides functional evidence linking phenolic metabolism to chemical defense against SCN. Together, these findings identify enhanced phenolic chemical defense as a major component of resistance to multiple SCN populations in wild soybean and highlight wild soybean as a valuable source of molecular and biochemical diversity for SCN resistance.

## Full Text Availability

The license terms selected by the author(s) for this preprint version do not permit archiving in PMC. The full text is available from the preprint server.

